# Death: Its Modes, Signs and Premonitions

**Published:** 1890-06

**Authors:** F. Bradnack


### DEATH: ITS MODES, SIGNS AND PREMONITIONS.

By F. BRADNACK, M. D.

*Death, as the Psalmist saith, is certain to all; all shall die.*—SHAKSPERE (K. HEN. IV.)

*Cinis et manes et fabula fies.*—PERSIUS.

*Death hath a thousand doors to let out life.*—MASSINGER.

*Accedit etiam mors, quæ quasi saxum Tantalo, semper impendet.*—CICERO.

*There are remedies for all things but death.*—CARLYLE.

*All our days travel toward death, and the last one reaches it.*—MONTAIGNE.

*By medicine life may be prolonged, yet death will seize the doctor too.*—CYMBELINE.

*Chaque instant de la vie est un pas vers mort.*—CORNEILLE.

---

"LET us not fight against facts," said Euripides, "for we can do them no harm." Now, *death is a fact.*

Furthermore, as, sooner or later, it is a fact which every man must personally experience, it may truly be said to be of universal interest, for death (as the Arab proverb declares) "is a black camel that kneels once at every man's gate."

It is the purpose of this article briefly to consider certain phenomena appertaining to death in their psychological, as well as in their physical and physiological aspects.

"To smell to a turf of fresh earth is," says old Fuller, "wholesome for the body; no less are thoughts of mortality cordial to the soul." Doubtless this is so. Nevertheless, we meet many persons who cannot bear even to think of death, much less to speak of it. The false hypersensitiveness of these people reminds one of the very modest young woman who could not endure to have a man's "naked eye" spoken of. Nevertheless, both naked eyes and death exist; and, as facts are facts, unquestionably the wisest plan is, not only to recognize them, but to look them squarely in the face, for necessity is coal black, and Death keeps no calendar. He of the Scythe will come sooner or later, and then physicians and remedies will be alike vain; for, as says the Persian poet, "of what use is camphor to a person struck by lightning?"

Faraday had a theory (and Flourens, I believe, held a similar one) that the natural limit of man's life is not seventy, but 100 years. He assumed that the length of an animal's life is five times as long as the period required for him to arrive at maturity. For instance, the dog takes two years to arrive at maturity, therefore, his limit should be ten years. Man, being twenty years in growing, lives 5 x 20=100. There is more than theory in this hypothesis, and the large number of individuals who exceed the traditional three score years and ten are facts toward its verification. Faraday also divided man's life into equal halves, growth and decline; and these into four stages, infancy, youth, virility, and age. *Infancy*, he said, extends to the twentieth year; *youth* to the fiftieth (because it is during this period that the tissues become firm, epiphyses united, etc.), *virility* from fifty to seventy-five, during which the organism remains complete, and at seventy-five *old age* begins, to last a longer or shorter time, according to the amount, so to speak, of reserve force then on hand, and also to the manner in which it is expended. It appears to the present writer that this is not only philosophical, but that it is also more or less backed up by facts. That very many human beings have lived far beyond seventy years is a matter of history and statistics. Galen is said to have reached 140 years, and during his long life to have composed over seven hundred essays on medical subjects. One Lawrence, an Englishman, by temperance and diet lived 140 years, and a man named Kentigern is recorded to have lived 180 years. This man never tasted either wine or spirits; was in the habit of frequently sleeping on the ground, and laboring hard. Henry Jenkins, of Yorkshire (a better authenticated case), was a poor fisherman, who ultimately became a beggar, lived uniformly on coarse and sparing diet, and attained to 169 years. But, perhaps, the most reliable case is the well-known historical one of old Parr, who reached the age of 153 years. It was certified that at the age of 120 he married a second wife, by whom he had one child. Being taken up to London "to see the King" by the Earl of Arundel, as a great curiosity, in his one hundred and fifty-second year, he soon thereafter died. The physician in charge made an autopsy in the case, and testified that the old fellow's death was occasioned by a change from a parsimonious to a plentiful diet. Old Parr was a farmer, and during all his life his habits had been entirely abstemious, his diet being solely milk, cheese, coarse bread, whey, and small beer. Perhaps, however, in his dotage he resembled old Ross, of Pottern, who lived till all the world was weary of him. In our own time we have many instances where the traditional seventy years have been exceeded. There is the late Emperor of Germany, whose age was ninety-two, and Lord Palmerston, who was eighty-three. Mr. Gladstone is now in his seventy-ninth year. Marshal McMahon is eighty-one; Meissonier is seventy-six; Robert Browning has just died at seventy-eight, and Dr. Oliver Wendell Holmes, who it is pleasant to think is still with us, is eighty.

It was the opinion of both Addison and Montaigne that nothing in history was more imposing or affecting than the accounts of the behavior of eminent persons in their dying hours. Regarded from the physician's point of view (*i. e.*, the physical), eminence or non-eminence is equally unimportant. It is somewhat surprising that so little of any value has been written on this momentous subject of death. Bichat endeavored to present a view of the changes in the system which are immediately concerned in the extinction of life. But even this was only one branch of the subject, and was far from exhausting it. But the state of knowledge compelled him to rest content with a general outline, although it was an outline drawn by a master's hand. Briefly stated, animal life and death may be defined as *fermentation* and *putrefaction*. Life is sustained by a process which may be called oxydization or combustion, or, broadly speaking, fermentation. To arrest this process, or too rapidly to accelerate it, is to cause death. Leibnitz, who had profound theories of life, had one of death also. He believed that generation is only the evolution of an animal already existing in form, and that death is merely the reënvelopment or involution of the same animal, which does not cease to subsist, but continues living. According to this view, birth and death are only changes in the order and adjustment of the principles of vitality—merely transformations. The eternal and imperishable germs of life neither cease nor begin. But what *does* begin and also perish is the organic machinery, of which these germs, so to speak, are the motive power. It is as if a man laid aside his clothes. Thus disrobed, he is naked, but is not, therefore, necessarily dead. So with the soul's corporeal environment, the body. To the man himself (the real man), the body is but as a suit of clothes. Such were the views of the philosophical Leibnitz ; and such also evidently was the view taken by two equally philosophical, if not equally scientific, minds, those of St. Paul and Shakspere.

It is the phenomena which precede, accompany, and follow this disrobing that we call physical death, which will concern us in the present paper.

Before proceeding further, it will be well to say a few words anent a popular error in connection with a series of events which precedes many cases where death is not sudden, known by the name of the "death agony." So far as this supposititious "agony" is concerned, its reality may be disputed. Its supposed existence furnishes a palmary instance of the fallacy of appearances, and of the folly of judging by them. Admitting a limited number of exceptions, to which all rules are liable, I firmly believe, judging from my own and others' medical observations at death-beds, that, as a rule, it is probably safe to say that *birth is a much more painful process than death ! A priori*, there exists no obvious, palpable reason, why death should be painful, much less why it should necessarily be an agonizing process. But, on the contrary, there seem to exist many excellent reasons why it should not be either one or the other. Firstly, a fallacy is involved in the very phrase " death agony." It is because in a certain proportion of cases dissolution is accompanied by visible spasm and distortion of the countenance, that this idea has obtained. Yet, it is as nearly certain as anything can be that these distortions of the facial muscles are not only painless, but take place unconsciously. They may be likened to the sudden flickerings in the dying flame of a candle, or to the irregular motions in the wheels of an engine, whose steam is being gradually withdrawn. Again, preceding death, in many instances, a comatose, or semi-comatose, stage supervenes, and it is altogether probable that more or less complete unconsciousness then prevails. There is also another species of evidence which is available on this subject. Many persons who were almost drowned have been resuscitated. Now, it is very extraordinary that all these, in relating their experiences, tell, practically, the same story. After a few moments of painful struggling, fear and anxiety pass away, and a state of tranquillity succeeds. They see visions of green fields (it is curious how invariably this optical illusion occurs), they, in some cases, hear pleasing music ; and, so far from being miserable, they experience a degree of ease and comfort of mind and body which is delightful. One proof that these descriptions are veracious, is the fact that in almost every instance where attempts at resuscitation prove successful, the resuscitated persons protest energetically against being brought back to life, and declare that restoration is accompanied by physical pain and acute mental misery. They reproach the agents bitterly, and upbraid them for withdrawing them from pleasing and paradisiacal scenes, to re-usher them into pain and wretchedness. I say it would be passing strange that the same series of phenomena should be so constantly reported if they were never experienced. There is no doubt that they are experienced, and they, therefore, confirm the proposition herein advanced, that death, as a rule, is by no means a painful process. In fact, the contrary theory is a pure assumption, and nothing else. "I wish," the famous Dr. Cullen, on his death-bed, faintly intimated to a friend, "I wish I had the power of writing or speaking, for then I would describe to you how pleasant a thing it is to die." And, Sir Walter Scott in his last moments exclaimed : "I feel as if I were to be myself again."

Especially in death at old age is the death-agony theory disproved. It is but rarely that we see a death which can be said to be the result of old age pure and simple. In those who live longest, some disease is usually developed ; but, occasionally, the body wears itself out, and without a malady or pain, sinks by slow degrees to decay. The organs have less life, the functions less vigor ; sight grows dim, hearing dull, touch obtuse ; in a word, vitality is reduced to a minimum. Life and death are, as it were, commingling. The strength is enfeebled, the bones are brittle, the muscles stiff, the ligaments dry, the arteries and veins atheromatous, and consequently inelastic ; the circulation is weak ; a slow decay has invaded every fiber. Lord Chesterfield, in this state of decrepitude, was unable to support the rapid motion of a carriage, and, when about to take an airing, said (in allusion to the slow pace at which they crept), "I am now going to the rehearsal of my funeral." In such cases there is not, when the end comes, even the semblance of any death agony. As a smouldering candle, which, toward the last, occasionally flickers into a momentary brightness, so do such lives go out. There is no sudden cessation of vitality, but a gradual, and often almost imperceptible extinction. So true is this, that it is often difficult to fix the precise moment of dissolution. I distinctly remember, in my own medical experience, one case of this sort, where, in answer to the friends' queries, I could only reply : "He may, or may not be dead ; but if life is not yet actually extinct, it is practically so," and desired them to cease all further attempts at restoration, and leave the patient in Nature's hands ; or, more properly speaking, in the hands of Him who is the Author of nature as well as of man. Dr. Baillie (very famous in his day as a practitioner in London, also a pupil of William Hunter,) once said, that all his observations of death-beds inclined him to believe that Nature intended we should go out of the world as unconscious as we came into it. "In all my experiences," he adds, "I have not seen one instance in fifty to the contrary." "To die by inches" has a dreadful sound, until we learn by observance that Time is the gentlest of all destroyers. The Latin proverb is true which avers that *The scythe of Time is more powerful than the club of Hercules.* But another expression so often used—"second childhood"—is a misnomer, because it involves a fallacy. There is no more resemblance between the first and so-called second childhood than there is in the vegetable world between immaturity and decay. There is an enormous difference between the prattle of youth and the prattle of old age ; the one is unripeness, the other decrepitude. Thus do we live in the midst of fallacies, which are perpetuated by the common speech.

All portions of the body, even the most solid, as the bones and teeth, are undergoing a continual but slow change. Their old cells are being destroyed, and new ones are being formed. Throughout the entire organism there is a continuous removal of effete tissue-matter, and a corresponding deposition from the circulating blood of new material. Every physical action is done at the expense of the organized structure ; in other words, in order to attain motive power, fuel must be burnt ; this fuel consists of minute particles of various organs. This burning is properly called *molecular* death ; but it is a death which, paradoxical as it may appear, is essential to life. Now, the stoppage of the heart's action, and that of the lungs also, (circulation and respiration,) is known as *somatic* death, or death of the body in its totality. Sir Thomas Watson remarks, in his Lectures on the Practice of Physic, that life rests on a tripod, whose three vital supports are the heart, the brain, and the lungs, and that through the impaired function of one or more of these organs, the tendency to death is expressed. It is not easy to give a definition of death, and it is still more difficult to give one of life. Definitions are proverbially difficult. However, broadly put, life, in its last analysis, and in its lowest forms, consists of functionally active protoplasm. The vital properties of protoplasm depend on a certain definite arrangement of certain molecules, and, as long as these molecules are so arranged, *life* is present. When, with man, death is said to have occurred, many of the phenomena associated with life may still be detected. The blood may yet move in the blood-vessels, heat may still be generated, glands may secrete, and the hair and nails may grow. General systemic, or somatic death can never exist except as a result of general molecular death. According to an idea, originating in the East, death in the bloom of youth was attributed to the attachment of some particular deity, who snatched his favorite to a better world. It was ascribed to Jupiter if caused by lightning, to the Nymphs if by drowning, to Aurora if it happened in the morning, and so on. During the most flourishing period of the arts of Greece, death was represented on tombs as a friendly genius with an inverted torch, and holding a wreath in his hand. The Hebrews had a fearful angel of death—Samaël—but he removes with a kiss those who die in early youth. The disgusting representations of death (a skeleton and a scythe) common amongst christian nations originated in the fourteenth century.

Bichat observed that animal life is made up of two orders of phenomena: (1) those of circulation and nutrition; (2) those that adjust the relations of the animal to its environment. He distinguishes organic life from animal life. Vegetables have the former, animals possess both. At the occurrence of death these two sorts of life do not disappear simultaneously. The animal life suffers the first stroke; the nervous system is first affected, and its operations retarded. Most commonly death occurs from some interference with the action of the vital organs, which, by the way, are not merely and only the brain, heart, and lungs, as stated by Sir Thomas Watson. The kidneys and the liver are certainly, in one sense, quite as vital organs as any of the three others. In zymotic fevers (*e. g.*, variola, scarlatina, typhus, and enteric fevers) the real cause of death may be defined by that much-abused term "blood poisoning." In these and such like diseases, the blood is truly poisoned, that is to say, chemically altered, and becomes, instead of a life-giving, a death-dealing fluid, carrying poison and destruction to every part.

Hemorrhage is a frequent cause of death. If the vital fluid is allowed to escape in large quantities from a severed artery or vein, pallor of the skin appears, coldness comes on, the breathing is sighing and intermittent; sight becomes dim; cold sweat covers the body; the *facies Hippocratica* soon manifests itself; the pulse grows weaker, and ere long the heart stops. "Sudden death," says a French writer,

"unconnected with outward and accidental causes, may occur in various ways. Very violent impressions on the feelings sometimes abruptly check the movements of the heart and produce a mortal swoon." Persons have been almost instantly killed not only by grief, but also by joy. Apoplexy and aneurism both kill, though in very different ways. There are also causes of sudden death which we do not yet fully understand, and therefore cannot explain. In many such cases an autopsy in no way discovers the true cause. Every physician knows this. Indeed, we may still exclaim with La Place, "What we know is little; what we do not know is immense."

### PREMATURE BURIAL.

Doubtless, in former times, before the heart's functions and the circulation of the blood were discovered, persons were occasionally buried alive, though many have been falsely stated to have been so buried. Some apparently authentic instances are on record. Facts collected by Bruhier and Lallemand furnish pretty strong rebutting evidence against sceptics in this matter. For instance: A rural guard, having no family, dies in a little village of Lower Charente (France). Hardly grown cold, his body is taken out of bed and laid on a straw ticking, covered with a coarse cloth. An old woman, a servant, is charged with the watch over the death-bed. At the feet of the corpse were a branch of boxwood, put into a vessel filled with holy water, and a lighted taper. Toward midnight the old watcher, being weary, fell asleep. Two hours later she awoke, surrounded by flames from a fire that had caught her clothes. She rushed out, crying for help, and the neighbors, running together at her screams, saw in a moment a naked spectre issue from the hut, limping and hobbling on limbs covered with burns. While the old woman slept, a spark had probably dropt on the straw bed, and the fire it kindled had aroused both the watcher from her sleep, and the supposed corpse from his seeming death. It is interesting to learn that, under proper care, he recovered from his burns, and grew sound and well again.

Philip Small, an English accoucheur, celebrated in his day, relates that on one occasion when he was summoned to perform the Cesarean operation, the by-standers, convinced that the woman was dead, urged him to proceed with it. "I supposed so, too," he says, "for I felt no pulse in the region of the heart, and a glass held over her face showed no sign of respiration." Then he plunged his knife into the body, and was cutting among the bleeding tissues, when the subject awoke from her swoon.

Vesalius, the eminent anatomist, opened an apparently dead body, in which the exposed heart was seen still beating; and the Abbe

Prevost, being struck down by apoplexy, was regarded as dead, but recovered consciousness when cut by a scalpel, only to die immediately afterward.

We occasionally meet even now-a-days, persons whose lives are rendered chronically miserable by the fear of being some day buried alive. I have myself met several such.

About the middle of the last century there died near Manchester, England, a maiden lady, Miss Bexwick, who had a great horror of being buried alive. To avoid this, she devised an estate to her medical attendant, Mr. Charles White, on condition that the doctor paid her a daily morning visit for twelve months after her decease. In order to to do this, it was requisite to embalm her, which he did. She was then placed in the attic of the old mansion in which she died, and in which the doctor took up his residence. Upon his leaving it, she was removed to the house erected by him in King street, Manchester, and which stood on the ground now occupied by the Town Hall. At the death of Mr. White, the doctor, she was sent to the Lying-in Hospital, where she remained till moved to her present resting-place, the Museum of Natural History, where the mummy is suspended in a case with a glass door.

### THE SIGNS OF IMPENDING DEATH.

These are many and variable. In no two instances can they be said to be identical, yet several are common to many cases. From a large number of statistics, it is now pretty well settled that the least mortality is during mid-day hours, and the greatest during the early morning hours—from three to six o'clock. Shakspere, who observed everything else, observed and recorded some of the premonitory signs of death also. In the account of the death of Falstaff they are accurately described—the sharpness of the nose (*his nose was as sharp as a pen*); the coldness of the feet, gradually extending upward (an almost infallible sign); the picking at the bed clothes (*carphologia*); and "the playing with flowers," a sign showing the weak and puerile state into which the once acute mind had fallen.

For some time before death, indications of its approach become apparent to observers. Speech grows thick and labored; the hands, if raised, fall instantly; the difficult respiration causes insufficient oxygenation of the blood; the heart loses its power to propel the blood into the extremities, which consequently become cold; a clammy moisture oozes through the pores of the skin; the voice grows weak, and husky or piping; the eyes begin to lose their lustre.

In death at old age there comes about gradually a dulling of all the bodily senses, and many of the mental faculties. The brain functions are imperfectly performed; memory fails (in varying degrees); imagination goes out like a candle; judgment wavers, and becomes an unreliable guide. The muscles and tendons get stiff, rendering digital operations which were previously easy, difficult or impossible. The voice breaks, and becomes a feeble, piping treble. The cords of the tabernacle are loosening. Solomon well describes this gradual approach of senile decay. Small noises irritate, (the grasshopper becomes a burden,) sight becomes dim (they who look out at the windows are darkened,) etc. Nutrition still goes on, but feebly, and with difficulty. Digestion is disturbed and impeded. The secretions are insufficient, or are vitiated, and not infrequently cease. Capillary circulation is clogged; this being due in part to the weakening of the heart-muscle, and in part to the calcareous deposits in the shrunken arteries. Finally, the central organ of the circulation comes to a stop, a full stop, and this stoppage means dissolution. This is the death of old age, but it is now comparatively rare. Few attain to it.

Among the several *minor signs* of impending death, these may be mentioned: (1) The embarrassment of speech; this, says Esquirol, is a mortal sign. (2) No wish to recover is doubtless often a sign of death's approach. (3) Loss of interest in things which formerly were interesting; to change one's habits smacks of death, says the Portuguese adage. Dr. George F. Shrady, of New York, a few years ago pointed out an up-and-down motion of the larynx (*Pomum Adami*) as a new sign of impending death. I have twice since been able to observe this sign, and can testify to its value, for in both instances its appearance was speedily followed by death.

### SIGNS OF ACTUAL DEATH.

It is the opinion of many of the laity, and even also of some physicians, that there exist no positive and indisputable signs of death. But this is an error. Doubtless many persons, due to this false belief, pass much of their time in fear and trembling, from the dreadful apprehension of the possibility of being buried alive. I have myself personally known two ladies who so feared and suffered. Such a chronic fear is surely worse than death itself; truly they die (as the poet says) a thousand deaths in fearing one. Now, *positive signs of the actual occurrence of death do exist*, and are known (or ought to be) to all educated physicians. There are at least five (5) signs which may fairly be characterized as indubitable, as, so to speak, pathognomonic of the last great human malady, death, namely: (1) cessation of circulation and respiration; (2) cooling of the body; (3) cadaveric rigidity; (4) resistance of the muscles to galvanic currents; (5) putrefaction.

Let us consider these in detail. (1) If the heart's action and also respiration have totally ceased for one hour, death has occurred. Dr. Taylor, in *Medical Juris.*, rightly says: "It is impossible to admit that the heart can remain for even half an hour in a state of inaction in a human being, and then spontaneously recover its activity."

In 1848 the Paris Academy of Sciences awarded a prize for a presentation of reliable signs of death. "Death," says the reporter of the commission, "is certain when positive cessation of the pulsation of the heart in the subject has been ascertained, which is immediately followed, if it has not been preceded, by cessation of respiration and of the functions of sensation and motion."

(2) *Cooling of the body.* The average temperature of the human body is about 99° F. This temperature is during life maintained irrespective of the temperature of the external air. When death occurs, the body begins to cool, and this process is continued until it cools down to the temperature of the surrounding atmosphere. The exact time required for this cooling varies, but it is usually accomplished in less than twenty-four hours, sometimes in fifteen hours, being accelerated or retarded according to circumstances.

(3) *Cadaveric rigidity.* This does not begin until several hours after death. Disappearance of general muscular contractility does not occur until still later, to be in its turn followed by putrefaction. In about six hours after death, and while the body is gradually cooling, a careful examination will show that the muscles of the limbs are becoming hard, the joints stiff, and the trunk unyielding. But this general contraction has succeeded to an opposite condition (which condition generally obtains at death),—*Relaxation*, shown by the dropping of the lower jaw, and other signs, at the moment of dissolution. But, after some hours of temporary rigidity, relaxation once more occurs, and, after this, putrefaction sets in.

(4) *Resistance of the muscles to galvanization.* The existence of this sign can be easily verified by the application of an ordinary galvanic battery. If life be present, when the current is passed through a muscle, the muscle responds by contracting. This contraction is proof that life exists, despite all appearances to the contrary. If it do not so respond, but remains rigid and immovable, life is extinct. There can be no disputing about this sign.

(5) *Putrefaction* generally commences about forty hours after death. Its first effects usually appear on the skin over the abdomen, in the form of a greenish discoloration, which gradually spreads over the whole surface of the body. Simultaneously certain moist and soft parts, as the eye and the lining of the mouth, begin to soften and decay; while, at the same time, the cadaverous odor is developed, which is at first a slightly moldy and fetid smell, but which soon becomes a pungent, mephitic, and ammoniacal stench. Gradually the flesh sinks in and grows watery, and one by one the various organs cease to be distinguishable. It is a somewhat curious fact that (in the female) the last organ to decay is the uterus. Putrefaction furnishes the sole *absolute* sign of death. It consists in a slow oxydation of the organic constituents of the body, brought about by the action of the air under the influence of bacterial organisms.

Besides the aforementioned, which may be called the *major signs*, there are also recognisable, or obtainable, soon after death, in many cases, certain other *minor* signs which are more or less diagnostic and reliable: (1) After death *the contractility of the skin is lost.* When a cut is made through the skin of a dead body, the edges of the wound collapse; while a similar lesion inflicted during life present an open or gaping appearance. (2) *The appearance of a green tint on the skin of the abdomen*, accompanied by separation of the epidermis, is a certain sign. (3) *When a thread is tied tightly around the finger*, if the person is living, the part beyond the ligature will become bluish-red in hue, while a narrow white ring will surround the finger where the ligature was applied; after the stoppage of the circulation no such appearance will be found. (4) If bright steel needles are thrust into the flesh during life, they will become tarnished from oxydation; *after death they will retain their brightness unchanged.* (5) If ammonia be injected under the skin during life, it will cause a deep red congestion of the part; *no such change occurs after death.* (6) The test of the *mirror held before the mouth and nostrils* (a test well known to Shakspere), is still available in some cases, but is not absolutely reliable. (7) At death *the pupil loses its mobility*, which mobility may be tested by passing a lighted candle close to the eyes; *the cornea loses its transparency*, also its lustre and sensibility. (8) There is developed the *facies Hippocratica.* (9) Later appear the marks of *post mortem lividity* (sugillations, livores), which are precursors of putrefaction. These are caused by the diffusion of the hematin of the blood from the red corpuscles.

Such are the signs of death.

( *To be concluded in July number.* )

